# Supplementary material for: Rapid turnover of life-cycle-related genes in the brown algae
Source: Genome Biol. 2019 Feb 14;20:35. doi: 10.1186/s13059-019-1630-6 (PMC6374913; doi:10.1186/s13059-019-1630-6)
Supplement: Supplementary file 3 — Protein alignments, corresponding Gblocks html file and CDS sequences for the lineage conserved gametophyte-biased genes. (ZIP 2109 kb) [file 13059_2019_1630_MOESM3_ESM.zip › Con_GA_alignments/Ec-05_000280.aa_ali.fasta-gb.htm]

Ec-05\_000280.aa.fasta.trans.aa\_ali.fasta


## Gblocks 0.91b Results

Processed file: **Ec-05\_000280.aa.fasta.trans.aa\_ali.fasta**  
Number of sequences: **2**  
Alignment assumed to be: **Protein**  
New number of positions: **833** (selected positions are underlined in blue)

```
                         10        20        30        40        50        60
                 =========+=========+=========+=========+=========+=========+
Ec-05_000280     MKSSRGPSIIF--PVANATAVVGKDEGDVEPSLVSQGHRTSCGKVTDGSPGRQSIGGDGQ
MSTRG.57900.1    -QLPRGDDVIRTAAVTDATEVVERSE-NVTSSLLWD--------VPEG------HGGKVP
                               ############                             #####


                         70        80        90       100       110       120
                 =========+=========+=========+=========+=========+=========+
Ec-05_000280     EMAFDGQYFHATAPKTCSPASAGRAVAQTLSQQGLVSSGFDNVNGALKIVTAAGAAAPGA
MSTRG.57900.1    RVARKEHRMVAVCPMEQEPGSVG-STPAALTET--------------EHVRLQQAVSSRR
                 #######################                          ###########


                        130       140       150       160       170       180
                 =========+=========+=========+=========+=========+=========+
Ec-05_000280     KEMGQDQPAGWYGPTASTATQSSLVCHGNNREKQPTSSCMNRAVRDNWGTVFVVGGGRRR
MSTRG.57900.1    KDTRAN--------PAGTDVGHRLSHARGERVASASGVKNDCVVRDSWGSVFIVGGGRRR
                 #              ################            #################


                        190       200       210       220       230       240
                 =========+=========+=========+=========+=========+=========+
Ec-05_000280     RRTVVRQRSVEVEETPAARRAQVEKERQERRKASLRRLMARKEQRALQQQSKMAIALNNR
MSTRG.57900.1    RRPAVRKRSSDAEETPAARRAQMEKQRQERRRSSLRRLMARKEQRAKQQAARRKRRAATA
                 #################################################           


                        250       260       270       280       290       300
                 =========+=========+=========+=========+=========+=========+
Ec-05_000280     RGEGKSPPKHPNAEAAIP------------------------------------------
MSTRG.57900.1    RAVAATPAVTADADKTVAGTEAVDRSSLATETTRHFSEPAEGGNDGAGTDGKERGVSTED
                 #############                                               


                        310       320       330       340       350       360
                 =========+=========+=========+=========+=========+=========+
Ec-05_000280     ----VPLFSLESGMSVDADPVMNDD--------------------------NMVDMDTEK
MSTRG.57900.1    EGAAGRKIVVESEQSRNNAAVVNDKKKVGTISGQIPCQTRRTMDISAVPRGSGVSLAAIT
                           ##############                             #######


                        370       380       390       400       410       420
                 =========+=========+=========+=========+=========+=========+
Ec-05_000280     AGTMGPGP------------------------------------DEWEEVGGQRYG----
MSTRG.57900.1    GGCKGSGSGEGSSDAAATASPAAAPAASINLVVRDKNDSCYSSGSESESVSGGDSSEEEC
                 #######                                                     


                        430       440       450       460       470       480
                 =========+=========+=========+=========+=========+=========+
Ec-05_000280     ------PIDDDC---------------------------------DS-------------
MSTRG.57900.1    LPVDASEICEDVDSDETASGSIARGGLEKEVVSGVTMHHPIPLGGDKEGGVSGCGVRAVS
                                                                             


                        490       500       510       520       530       540
                 =========+=========+=========+=========+=========+=========+
Ec-05_000280     --DNTEGSGATLELQG--------------------------------------------
MSTRG.57900.1    GRSGSSGYGASLEDAGDCRSAVTHDARPGNNVDGDPGFESSIASGETAIVRRGGGSPCVP
                       ##########                                            


                        550       560       570       580       590       600
                 =========+=========+=========+=========+=========+=========+
Ec-05_000280     ------------------------------------PLHVQPLAETR----QAEHRSAIR
MSTRG.57900.1    IRSACAWDIGHLNHGVPASATTARSLHHEYYSSKYAAQEVQAVAVVEGAATRAEEASYLR
                                                                     ########


                        610       620       630       640       650       660
                 =========+=========+=========+=========+=========+=========+
Ec-05_000280     STSCGALG----------------------------------------------------
MSTRG.57900.1    STSTIVDASCLRDYFPKNNQDEAMTGTLPSKSDGSDSGMESEEYYKRGDDGAADLVCGER
                 ###                                                         


                        670       680       690       700       710       720
                 =========+=========+=========+=========+=========+=========+
Ec-05_000280     -----------------------------------------TVTPQLPNCDADETKQGRN
MSTRG.57900.1    SQRGRANDGSLEFPGPLDVQPLIGIYPTKASLASENGGSDIQEDQPLLTHSAGISEAVRI
                                                               ##############


                        730       740       750       760       770       780
                 =========+=========+=========+=========+=========+=========+
Ec-05_000280     PGLIASANGIVSVSDVDL----AGPRRPTGLSPSAPTSASPRLSVPLLSMDLCSPS----
MSTRG.57900.1    SARFAPDSRVNTTSCASCAGGSRNPKTPGDLFPFTSVPISPPPSIPLESRKTCSSLRPHS
                 ##############          ##############################      


                        790       800       810       820       830       840
                 =========+=========+=========+=========+=========+=========+
Ec-05_000280     -------------------------DGGTGNGCAVVGVEPLLPEPPAPRMYQPEGRNQAA
MSTRG.57900.1    ADHVAAAEIMEEHRGVQQPLEQGVMKGDDGFGAVATSAHEQVPDPPAMEARTHGCRNEII
                                                           ###############   


                        850       860       870       880       890       900
                 =========+=========+=========+=========+=========+=========+
Ec-05_000280     AGRKEGTGDNWKAPINSKSQECRDVSKQS------YSAGDAIVDNTSARFTVEPPDAFTA
MSTRG.57900.1    PRKQDLGSRGNSAGARTRGEPRHDLESPDPDFPKITHMAKVKRDGRDSQHVASADCSMTM
                                                                             


                        910       920       930       940       950       960
                 =========+=========+=========+=========+=========+=========+
Ec-05_000280     VPQGRRPRRSGSLTTPPVMGTGDDGSKATCLQAEVPETPGGAAGGDVAGHFEPVEKVPSS
MSTRG.57900.1    IDRASTTYAKASRQTFLVTSEERQGRKGSAILQLPEPQVGASDDVSTGSPFKSGDGIAES
                            ################                       ##########


                        970       980       990      1000      1010      1020
                 =========+=========+=========+=========+=========+=========+
Ec-05_000280     ------------NTLATYTDCFPRFAAILSAHHNGRALTRQADCRSLPLTSKILSRGRAG
MSTRG.57900.1    NVDAAVGTSHSDVDSIRFVKCYPRFAAILTAYHNGRALTRQADFRSLPLTSKIISRGMAA
                                     ########################################


                       1030      1040      1050      1060      1070      1080
                 =========+=========+=========+=========+=========+=========+
Ec-05_000280     AGGS--AANRRGIKGLGLTGAEEEACFHWQWKLYAIHVRVIKDYAAVFSYGDGWSGNGTS
MSTRG.57900.1    GGSSRVIAGHRGGIGSTLTAAEEEACLHLQWKLYTIHVRVIKDYATVFSYGDGWSTDGAA
                 ####   #####################################################


                       1090      1100      1110      1120      1130      1140
                 =========+=========+=========+=========+=========+=========+
Ec-05_000280     DDSNSLVYSGDSSALHYRVNSKARPEVYNIVTDVLNNRCQQWEELPTGLGLKTTWNLLWT
MSTRG.57900.1    DEPNSLVYSGDSTALHYRVNSKARPEVYNIVTDVLNNRCEQWEELPTGLGLKTTWNLLWT
                 ############################################################


                       1150      1160      1170      1180      1190      1200
                 =========+=========+=========+=========+=========+=========+
Ec-05_000280     WSKPHIQYSSLLTWQKVNHFPNSRELTRKDLLNKHVSRFMAPGGRLAKEFHIMPQTFVLP
MSTRG.57900.1    WSKPHIRYSSLLTWQKVNHFPNSRELTRKDLLNKHVSRFMAPGGRLGKEFHIMPQTFVLP
                 ############################################################


                       1210      1220      1230      1240      1250      1260
                 =========+=========+=========+=========+=========+=========+
Ec-05_000280     HEFTLFVSAFTSS-------------------------------------------SGTR
MSTRG.57900.1    HEFTLFVTAFTSLGRTQPHRRGNTTSGDLDGNGMLSGADDDEDDGFTCGKSGARRGGGKR
                 ############                                             ###


                       1270      1280      1290      1300      1310      1320
                 =========+=========+=========+=========+=========+=========+
Ec-05_000280     ENLWIMKPVGMSRGRGIRLIDDIKDICYADKVVLQRYIGNPLLLDGYKFDLRLYVLVTSF
MSTRG.57900.1    ENLWIMKPVGMSRGRGIRLIDDIKNICYADKVVLQRYIGNPLLLDGYKFDLRLYVLVTSF
                 ############################################################


                       1330      1340      1350      1360      1370      1380
                 =========+=========+=========+=========+=========+=========+
Ec-05_000280     NKLEAFIYKEGFARLSTHRYEKGAIDNRFIHLTNSSIQRLNEAGAARDSPLNQAKTSEAG
MSTRG.57900.1    NKLEAFIYKEGFARLSTHRYKKGDIDNRFIHLTNSSIQRLNEAGAARDSPLHRAKTSEAG
                 ############################################################


                       1390      1400      1410      1420      1430      1440
                 =========+=========+=========+=========+=========+=========+
Ec-05_000280     GTKTTLSYLWRRLAASGVDVGALWAEICSVVVKSLVCVESSIPNQPNSFELFGYDVLIDD
MSTRG.57900.1    GTKTTLSYLWRRLAASGVDVGALWAGICSLVVKSLVCVERSIPNQPNSFELFGYDVLIDD
                 ############################################################


                       1450      1460      1470      1480      1490      1500
                 =========+=========+=========+=========+=========+=========+
Ec-05_000280     RLRPWLIEVNASPSMARDTHVD---------------------RQKLVDILGRRLKETEH
MSTRG.57900.1    RLRPWLIEVNSSPSMARDTHVDRQVKEALIFETVHLVDPLPFDRQKLVDILGRRLSETQN
                 ######################                     #################


                       1510      1520      1530      1540      1550      1560
                 =========+=========+=========+=========+=========+=========+
Ec-05_000280     SKTRAFKQRHYPHHHQQAFDTTEVQGDARAGDN---VYPSPSAKASA------SREH--Q
MSTRG.57900.1    SKGRTFKQWHHHRQRQPQRSQSQQPFGSETGTSIGRESPSPSARASTREEHHTGEEHRQQ
                 ################                                           #


                       1570      1580      1590      1600      1610      1620
                 =========+=========+=========+=========+=========+=========+
Ec-05_000280     RPRRTRGEADLESDLDQILRGRRPRQVGELPARMGNFVRLCPGTAAHRRARRLRRKVIRP
MSTRG.57900.1    RRRTSKEEAELEKDLDDILCGRRPRQVGEKPARMGNFVRLCPGTTAYRHARRLRRKVIRP
                 ############################################################


                       1630
                 =========+=====
Ec-05_000280     QPGDDDDPTGSK--R
MSTRG.57900.1    RPGDDGDDDGPILNT
                 ##########
```

```
Parameters used
Minimum Number Of Sequences For A Conserved Position: 2
Minimum Number Of Sequences For A Flanking Position: 2
Maximum Number Of Contiguous Nonconserved Positions: 8
Minimum Length Of A Block: 10
Allowed Gap Positions: None
Use Similarity Matrices: Yes
```

```
Flank positions of the 20 selected block(s)
Flanks: [15  26]  [56  83]  [110  121]  [136  151]  [164  229]  [241  253]  [311  324]  [354  367]  [487  496]  [593  603]  [707  734]  [745  774]  [823  837]  [912  927]  [951  960]  [981  1024]  [1028  1212]  [1258  1462]  [1484  1516]  [1560  1630]  

New number of positions in Ec-05_000280.aa.fasta.trans.aa_ali.fasta-gb:  833  (50% of the original 1635 positions)
```
